# Parental warmth, adolescent emotion regulation, and adolescents’ mental health during the COVID-19 pandemic

**DOI:** 10.3389/fpsyg.2023.1216502

**Published:** 2023-09-01

**Authors:** AnnaMaria Boullion, Linnea B. Linde-Krieger, Stacey N. Doan, Tuppett M. Yates

**Affiliations:** ^1^Department of Psychology, University of California, Riverside, CA, United States; ^2^Department of Family and Community Medicine, University of Arizona, AZ, United States; ^3^Claremont McKenna College, Claremont, CA, United States; ^4^City of Hope National Medical Center, Duarte, CA, United States

**Keywords:** adolescence, COVID-19, emotion regulation, longitudinal, mental health, multiple mediation

## Abstract

**Introduction:**

The United States (U.S.) Surgeon General Advisory has characterized the COVID-19 pandemic as a youth mental health crisis. Thus, elucidating factors affecting adolescents’ mental health during the pandemic is important for supporting youth through current and future challenges. Parenting influences adolescents’ ability to cope with stressors, and emotion regulation strategy use may underlie these effects.

**Methods:**

This longitudinal study of 206 adolescents (49% female; 46.6% Latine) from the U.S. evaluated pathways from perceived parental warmth and affection at age 12 to changes in adolescents’ internalizing and externalizing problems from before the pandemic (age 14) to the initial phase of the U.S COVID-19 pandemic in Spring 2020 (age 15) through adolescents’ pre-pandemic cognitive reappraisal and expressive suppression emotion regulation strategy use at age 14.

**Results:**

Parental warmth and affection predicted decreased internalizing, but not externalizing, problems during the initial phase of the pandemic, and this effect was explained by adolescents’ reduced reliance on expressive suppression as an emotion regulation strategy.

**Conclusion:**

These findings illuminate parenting and emotion regulation strategy selection as modifiable processes to support adolescents’ mental health in this crisis and beyond.

## Introduction

The COVID-19 pandemic negatively affected the physical, economic, and psychological well-being of individuals across the world. In addition to global grieving over lost lives, efforts to prevent the spread of COVID-19 (e.g., isolation, remote learning; Park et al., 2020) negatively impacted mental health (Hertz-Palmor et al., 2021), particularly for adolescents (Samji et al., 2022). Longitudinal studies have documented significant increases in adolescents’ internalizing symptoms (e.g., anxiety, depression; Ravens-Sieberer et al., 2022) and externalizing symptoms (e.g., attention problems, rule-breaking; Rosen et al., 2021) over pre-pandemic levels. Informed by these patterns, the United States (U.S.) Surgeon General issued an advisory underscoring the youth mental health crisis precipitated by the COVID-19 pandemic on December 6th, 2021, and encouraged research to understand changes in adolescents’ mental health and identify key opportunities for support (U.S. Surgeon General’s Advisory, 2021).

Despite trends toward increased mental health difficulties among adolescents during the COVID-19 pandemic, marked individual differences remained. Research to identify mechanisms driving these individual differences will inform efforts to support adolescents’ navigation of future challenges. Thus, this investigation drew on an ongoing longitudinal study to evaluate prospective relations between warmth and affection in the parent–child relationship at age 12 and adolescents’ mental health during the first phase of the U.S. COVID-19 pandemic 3 years later (i.e., spring 2020; age 15) beyond adolescents’ pre-pandemic problems at age 14. A mediation analysis evaluated the extent to which hypothesized positive effects of parental warmth and affection on adolescents’ mental health during the pandemic would be explained by adolescents’ pre-pandemic emotion regulation strategy use at age 14.

### Parenting and adolescent mental health

A host of parenting qualities and practices (e.g., hostility, demandingness, supportiveness, responsiveness) influence youth’s internalizing and externalizing behavior problems (Pinquart, 2017a, b). Parental warmth and affection, which is linked to parental responsiveness (Khaleque and Rhoner, 2002), is a particularly important aspect of parenting for understanding adaptive adjustment across adolescence (Alcaide et al., 2023), demonstrating positive relations with adolescent adjustment across diverse cultural groups (Khaleque, 2013), and as relevant to adolescents’ capacities for socioemotional resilience in contexts of adversity (Masten et al., 2004). For example, in a study of adolescents’ anxiety pre- and post-hurricane Katrina, Costa et al. (2009) found that adolescents who reported their parents engaged in low communication, acceptance, and high control prior to Katrina showed elevated rates of anxiety in response to the hurricane as compared to their peers who reported their parents provided more communication, warmth, and sensitivity.

Parenting effects may be magnified in stressful contexts, especially during the COVID-19 pandemic when lockdown restrictions limited developmentally normative peer connections (Hartup, 1989) and heightened the salience and frequency of parent-adolescent contact. During the COVID-19 pandemic, cross-sectional data showed that positive parenting (e.g., emotion socialization, supportive responses to pandemic-related reactions) has the potential to mitigate negative relations between COVID-19 stressors and adolescents’ adjustment (Cohodes et al., 2021). However, both longitudinal studies that include pre-pandemic controls and process-oriented studies that evaluate potential mediating mechanisms underlying these effects remain rare. The current study filled these gaps by evaluating prospective relations from early adolescents’ reports of parental warmth and affection at age 12 to adolescents’ COVID-19 mental health symptomatology 3 years later during the spring of 2020 (i.e., age 15) as related to adolescents’ pre-pandemic emotion regulation strategy use at age 14.

### Parenting and adolescent emotion regulation

Emotion regulation involves monitoring, appraising, and modifying one’s emotional reactions to upregulate, downregulate, or maintain emotional states to achieve a desired regulatory goal (Gross, 2015). Effective deployment of emotion regulation strategies requires adequate emotional knowledge (Denham et al., 2015) and confidence in one’s ability to manage emotions, both of which develop in the context of parent–child relational exchanges (Brumariu, 2015). Thus, emotion regulation is a central mechanism by which parenting may influence youth adjustment. Further, as with parenting effects in contexts of adversity, emotion regulation takes on heightened adaptive significance in risky contexts.

Among the many strategies used to regulate emotions (McRae and Gross, 2020), antecedent-focused *cognitive reappraisal* (CR; i.e., altering one’s thoughts about an emotionally evocative stimulus to change its emotional impact; John and Gross, 2004) is more often associated with positive adjustment outcomes (Aldao et al., 2010) than response-focused *expressive suppression* (ES; i.e., inhibiting emotionally expressive behaviors; Gross, 1998), which is commonly associated with negative adjustment outcomes (Dryman and Heimberg, 2018). Among adolescents, CR is related to greater life satisfaction, perceived social support, and positive affect (Verzeletti et al., 2016), as well as fewer internalizing problems (Garnefski et al., 2005) and better emotional recovery after a social stressor (Shapero et al., 2019). In contrast, adolescents’ ES is related to strained social interactions (Butler et al., 2003), PTSD symptoms (Zhou et al., 2017), and internalizing problems (Balan et al., 2017).

A sizable body of evidence indicates that parenting behaviors influence the degree to which youth engage specific emotion regulation strategies. For example, in a sample of young children, Gunzenhauser et al. (2014) found that parents’ support of their child’s emotional experiences coupled with their own use of CR predicted their child’s use of CR. In contrast, parents who were unsupportive (i.e., minimizing or punitive reactions) of their child’s emotional experiences and who used ES to manage their own emotions had children who were more likely to use ES. Extending to early adolescence, Jaffe et al. (2010) found that warm and affectionate parenting as reported by youth in early adolescence (9–12 years old) predicted adolescents’ greater concurrent use of CR, whereas less warmth and affection was associated with greater use of ES. Informed by these prior studies, we hypothesized that parental warmth and affection in early adolescence would be related to adolescents’ later use of more CR and less ES emotion regulation strategies.

### Parenting, emotion regulation, and adolescent mental health

Emotion regulation skills are a salient mechanism undergirding parenting effects on adolescents’ socioemotional adaptation (Sheppes and Gross, 2011). These effects may be especially pronounced in adolescence because it is a uniquely vulnerable time for socio-emotional development (Rapee et al., 2019), as well as in the context of the COVID-19 pandemic because it was a stressor for children and families. Indeed, adaptive strategies, such as CR, typically decrease across early adolescence with less adaptive strategies, such as ES, taking hold during mid-adolescence (Gullone et al., 2010; Zimmermann and Iwanski, 2014). Coincident with increasing reliance on potentially problematic emotion regulation strategies, adolescents also develop heightened levels of internalizing (McLaughlin et al., 2011) and externalizing (Compas et al., 2017) problems, particularly in stressful contexts (Larson et al., 2002), such as the COVID-19 pandemic (Park et al., 2020).

A handful of studies have tested mediating relations from elements of parenting associated with warmth and affection to adolescent mental health problems via CR and/or ES emotion regulation strategies. For example, Ogbaselase et al. (2020) found that adolescents who reported family exchanges characterized by high levels of negative emotion, low levels of positive emotion, and low parental warmth endorsed lower reliance on CR and greater use of ES which, in turn, predicted elevated depressive symptoms. Likewise, negative parenting practices, such as inconsistent discipline, corporal punishment, and poor monitoring, are linked to adolescents’ higher internalizing difficulties through their greater use of ES (Balan et al., 2017). Regarding positive parenting quality, parent’s supportive responses to adolescents’ negative emotions are linked to better wellbeing through emotion regulation strategies, such as ES and CR (Ding et al., 2022). Walton and Flouri (2010) found that adolescents who reported higher parental warmth and affection endorsed fewer emotion regulation difficulties and, by extension, fewer conduct problems than those who reported lower parental warmth and affection. Similarly, adolescents who reported high autonomy support from their mother in early adolescence engaged in less ES, and, ultimately, experienced fewer depressive symptoms, as compared to adolescents who reported lower levels of maternal autonomy support and higher rates of ES strategy use (Brenning et al., 2015). Therefore, we hypothesized that parental warmth and affection during early adolescence would be related to adolescents’ increased use of positive emotion regulation strategies, such as CR, and decreased use of problematic strategies, such as ES, in ways that would protect and promote adolescents’ mental health during the COVID-19 pandemic.

### The current study

Recent data suggest that close and secure caregiver-child relationships promote better-than-expected mental health responses to the COVID-19 pandemic (Coulombe and Yates, 2021). Likewise, some research has shown that adolescents’ pre-pandemic emotion regulation difficulties predicted more mental health problems during the pandemic (Breaux et al., 2021). Extending these investigations, the current study offered a novel test of theoretically specified mediating relations from parental warmth and affection at age 12 to fewer internalizing and externalizing problems during the initial phase of the U.S. COVID-19 pandemic at age 15 via adolescents’ pre-pandemic emotion regulation strategy use at age 14. Importantly, this investigation examined both internalizing *and* externalizing problems simultaneously with both CR *and* ES emotion regulation strategies, whereas prior studies have typically focused on either ES *or* CR (Balan et al., 2017) as related to either internalizing *or* externalizing symptoms (e.g., Walton and Flouri, 2010; Weissman et al., 2021). We hypothesized that parental warmth and affection at age 12 would be associated with adolescents’ later use of more frequent CR and less frequent ES emotion regulation strategies at age 14. In turn, we predicted that pre-pandemic reports of more CR and less ES would be related to fewer internalizing and externalizing problems in the context of the COVID-19 pandemic 1 year later over and above pre-pandemic symptomatology.

In addition to controlling for prior levels of internalizing and externalizing problems, this three-wave longitudinal investigation considered adolescents’ sex assigned at birth, ethnicity-race, family income-to-needs, and contemporaneous exposure to stressful life events during the pandemic as potentially salient influences on the hypothesized relations. Extant literature points to significant sex differences in mental health problems as related to parenting (Lansford et al., 2014), emotion regulation (Nolen-Hoeksema, 2012), and the COVID-19 pandemic (Magson et al., 2021). Moreover, given documented disparities in COVID-19 experiences (i.e., morbidity and mortality rates) across ethnic, racial, and economic groups (Karmakar et al., 2021), as well as in parenting influences on adolescent adjustment (Williams and Merten, 2014), we included these sociodemographic characteristics as covariates. Due to the negative influences of stressors related to COVID-19 on the parent–child relationship and adolescents’ mental health during the pandemic (Achterberg et al., 2021), we also controlled for adolescents’ contemporaneous reports of COVID-19 stressors (e.g., missed or canceled events, parental job loss, death or serious illness of a family member).

## Method

### Participants

The current sample was drawn from an ongoing study following 250 caregiver-child dyads every 1–2 years from preschool through late adolescence. The current analyses included the 206 dyads that completed one or more assessments at ages 12, 14, and/or 15. Participating caregivers at age 12 were biological mothers (92%), biological fathers (3%), adoptive mothers (2.5%), and other female extended kin (2.5%). Adolescents completed assessments at age 12 (*N* = 201; *M_age_W1_* = 12.25; *SD* = 0.35), 1 year prior to the onset of COVID-19 pandemic at age 14 (*N* = 160; *M_age_W2_* = 14.19; SD = 0.28), and/or during the first 2 months of the U.S. national emergency declaration in the spring of 2020 at age 15 (*N* = 157; *M_age_W3_* = 15.22; *SD* = 0.57). The sample was diverse with respect to sex (49% female sex assigned at birth, 51% male sex assigned at birth), ethnicity and race (46.6% Latine, 24.3% multiethnic/racial, 18.4% Black, 10.2% white, 0.5% Asian), and income (23.5% qualified for government assistance). Data for adolescents who completed one or more study waves were included in these analyses. Of the 206 participating adolescents, 173 (83.9%) participated in two or more data waves.

### Procedures

Flyers inviting participation in a longitudinal study of children’s learning and development were distributed to community-based childcare centers in Southern California. Families were screened to ensure the child was between the ages of 3.9 and 4.6 months, proficient in English, and not diagnosed with a developmental disability at the time of the first assessment. Several years later, these same children completed a variety of measures, which were administered in-person at age 12, via telephone at age 14, and using an on-line survey at age 15 during the initial months of the COVID-19 pandemic when stay-at-home orders were in effect. Informed consent was obtained from the legal guardian and informed assent was collected from the participating adolescent at each wave. Adolescents were compensated $10–25 per assessment hour across waves. All procedures were approved by the human research review board of the participating university.

### Measures

#### Parental warmth and affection

At age 12, adolescents completed the short form of the Parental Acceptance and Rejection Questionnaire (PARQ-SF; Rohner et al., 1978). Adolescents reported their perception of their caregiver’s warmth and affection across 8 items (e.g., My caregiver makes it easy for me to tell them things that are important to me) on a scale from 1 (*almost never true*) to 4 (*almost always true*). The average item score was used for these analyses. The PARQ-SF has been shown to be a reliable measure across diverse ethnic and cultural groups (Khaleque and Rohner, 2002), including in the current study (alpha = 0.74).

#### Emotion regulation

At age 14, adolescents completed the 10-item Emotion Regulation Questionnaire for children and adolescents (ERQ-CA; Gross and John, 1998). Adolescents responded to six items assessing their tendency to use CR (e.g., When I want to feel happier, I think about something different) and four items assessing their tendency to use ES (e.g., When I am feeling happy, I am careful not to show it) on a scale from 1 (*not at all true for me*) to 3 (*really true for me*). The average item score was used for these analyses. This measure has shown good internal consistency across a 12-month period (Gullone and Taffe, 2012), and reliabilities for both the CR (alpha = 0.78) and ES (alpha = 0.66) scales were acceptable in this sample.

#### Behavior problems

At ages 14 (1 year prior to the U.S. national emergency declaration) and 15 (in spring of 2020), adolescents completed the Youth Self Report (YSR; Achenbach and Edelbrock, 1991). The YSR is a 112-item questionnaire asking adolescents to respond to statements about their behaviors on a 3-point-likert scale ranging from 0 (*not true*) to 2 (*very true/often true*). At age 14, adolescents reported their problem behaviors within the prior 6 months. However, to capture problems in response to the initial COVID-19 crisis, adolescents were asked to report on their behavior problems within the previous 2 weeks at the spring 2020 COVID-19 assessment. The internalizing and externalizing broadband *t*-scores from the YSR were used in these analyses. The internalizing scale incorporated 31 items tapping anxiety, depression, and somatic complaints (e.g., I worry a lot; alphas = 0.90 and 0.91 at ages 14 and 15, respectively). The externalizing scale included 32 items about rule-breaking, hyperactivity, and aggressive behaviors (e.g., I have a hot temper; alphas = 0.90 at both ages 14 and 15).

#### Family income-to-needs

At age 12, family financial resources were determined based on the caregiver’s reported household income. Caregivers described all financial contributions to the household during the preceding 12-month period (e.g., salary, child support). This figure was divided by the appropriate poverty threshold for a one- or two-parent household and the number of dependent children in the home (U.S. Census Bureau, 2016) to yield the continuous income-to-needs ratio that was used in all analyses.

#### Stressors related to COVID-19

At age 15, an adapted version of the Adolescent Life Events scale (ALEQ; Hankin and Abramson, 2002) assessed adolescents’ exposure to stressors related to COVID-19 during the initial phase of the pandemic. Adolescents reported either 0 (*no*) or 1 (*yes*) regarding whether they had experienced 22 negative events during the preceding 2 weeks, which were adapted to capture specific stressors related to COVID-19 (e.g., Has your caregiver tested positive for coronavirus/COVID-19?). Stress related to COVID-19 was indicated by the total number of stressful life events endorsed by adolescents.

### Data analytic plan

Descriptive and bivariate analyses were conducted using IBM SPSS Statistics (Version 27). A multivariate analysis of variance (MANOVA) evaluated group differences across study variables as a function of adolescents’ sex assigned at birth, ethnicity-race, and their interaction. The hypothesized parallel mediation model was evaluated using the Lavaan package (Rosseel, 2012) in RStudio 4.1.0 (RStudio Team, 2021). All analyses controlled for adolescents’ sex, ethnicity and race, family income-to-needs, stressors related to COVID-19, and pre-pandemic symptomatology. A sensitivity analysis evaluated this same model using only the 122 participants who provided complete data at all three waves of the study.

Inspection of missing data patterns revealed that 5 (2.4%) adolescents were missing data on parental warmth and affection because they did not complete the age 12 assessment, and an additional 12 (5.8%) adolescents did not complete the PARQ due to time constraints. Five families (2.4%) were missing income-to-needs data because they did not complete the age 12 assessment and an additional 5 (2.4%) were missing due to insufficient information provided. At age 14, 46 (22.3%) adolescents did not have data on the ERQ or YSR because they did not complete the age 14 assessment. At age 15, 49 (23.8%) adolescents were missing data on internalizing and externalizing problems because they did not complete the COVID-19 assessment, and one (0.5%) additional adolescent did not complete the YSR during the assessment. Little’s (1988) MCAR test indicated data were missing at random, χ^2^(169) = 192.96, *p* = 0.100, supporting the use of full information maximum likelihood (FIML) to handle missing data (Schafer and Graham, 2002).

## Results

### Descriptive and bivariate analyses

Table 1 depicts descriptive and bivariate relations among study variables. Paired-samples *t*-tests utilizing listwise deletion revealed significant increases in adolescents’ internalizing and externalizing problems from pre-pandemic (*M*_internalizing_ = 45.91; *SD* = 10.46; *M*_externalizing_ = 44.31; *SD* = 10.70) to COVID-19 (*M*_internalizing_ = 51.26; *SD* = 11.85; *M*_externalizing_ = 48.58; *SD* = 10.52) reports (*t*_internalizing_ [133] = −3.78, *p* < 0.001; *t*_externalizing_ [133] = −2.76, *p* = 0.007). A MANOVA tested whether there were differences by sex (i.e., females, males), ethnicity-race (i.e., white, Black, Latine, multi-ethnic/racial/other), or their interaction across all nine study variables (i.e., family income-to-needs, parental warmth, ES and CR internalizing and externalizing symptoms at ages 14 and 15, and stressors related to COVID-19). Based on the 122 participants with complete data at all waves, the MANOVA revealed no significant main effects by adolescents’ sex [*F*(9, 106) = 1.73, *p* = 0.091; Wilks’ λ = 0.872], ethnicity-race [*F*(27, 310) = 0.72, *p* = 0.849; Wilks’ λ = 0.838], nor their interaction [*F*(27, 310) = 1.31, *p* = 0.144; Wilks’ λ = 0.730].

**Table 1:** Descriptive statistics and bivariate correlations of study variables.

	1	2	3	4	5	6	7	8	9
Family income-to-needs ratio (age 12)	–								
Parental warmth/affection (age 12)	0.077	–							
Cognitive reappraisal (age 14)	0.055	0.195^*^	–						
Expressive suppression (age 14)	0.062	−0.177^*^	0.159^*^	–					
Pre-COVID-19 internalizing problems (age 14)	−0.049	−0.144	−0.044	0.167^*^	–				
Pre-COVID-19 externalizing problems (age 14)	0.057	−0.046	0.046	0.253^**^	0.660^**^	–			
COVID-19 internalizing problems (age 15)	0.019	−0.016	−0.055	0.315^**^	0.098	0.087	–		
COVID-19 externalizing problems (age 15)	0.025	−0.096	−0.018	0.251^**^	0.060	0.074	0.676^**^	–	
COVID-19 related stressors (age 15)	0.204^*^	0.011	0.076	−0.001	0.053	0.007	0.336^**^	0.198^*^	–
*M*	2.35	3.67	2.18	1.82	45.93	44.31	51.26	48.58	2.51
*SD*	1.5	0.4	0.5	0.5	10.5	10.7	11.8	10.5	2.8

Note: *p < 0.05 level. **p < 0.001.

Bivariate analyses showed parental warmth and affection at age 12 was positively related to CR and negatively related to ES emotion regulation strategy use at age 14, though CR and ES emotion regulation strategies were positively correlated with another. Adolescents’ internalizing and externalizing problems were positively and concurrently related at both ages 14 and 15. ES was positively related to internalizing and externalizing problems at both ages 14 and 15. Family income-to-needs at age 12 and behavior problems at age 15 were positively related to stressors related to COVID-19.

### Mediation analysis

**Figure 1:**
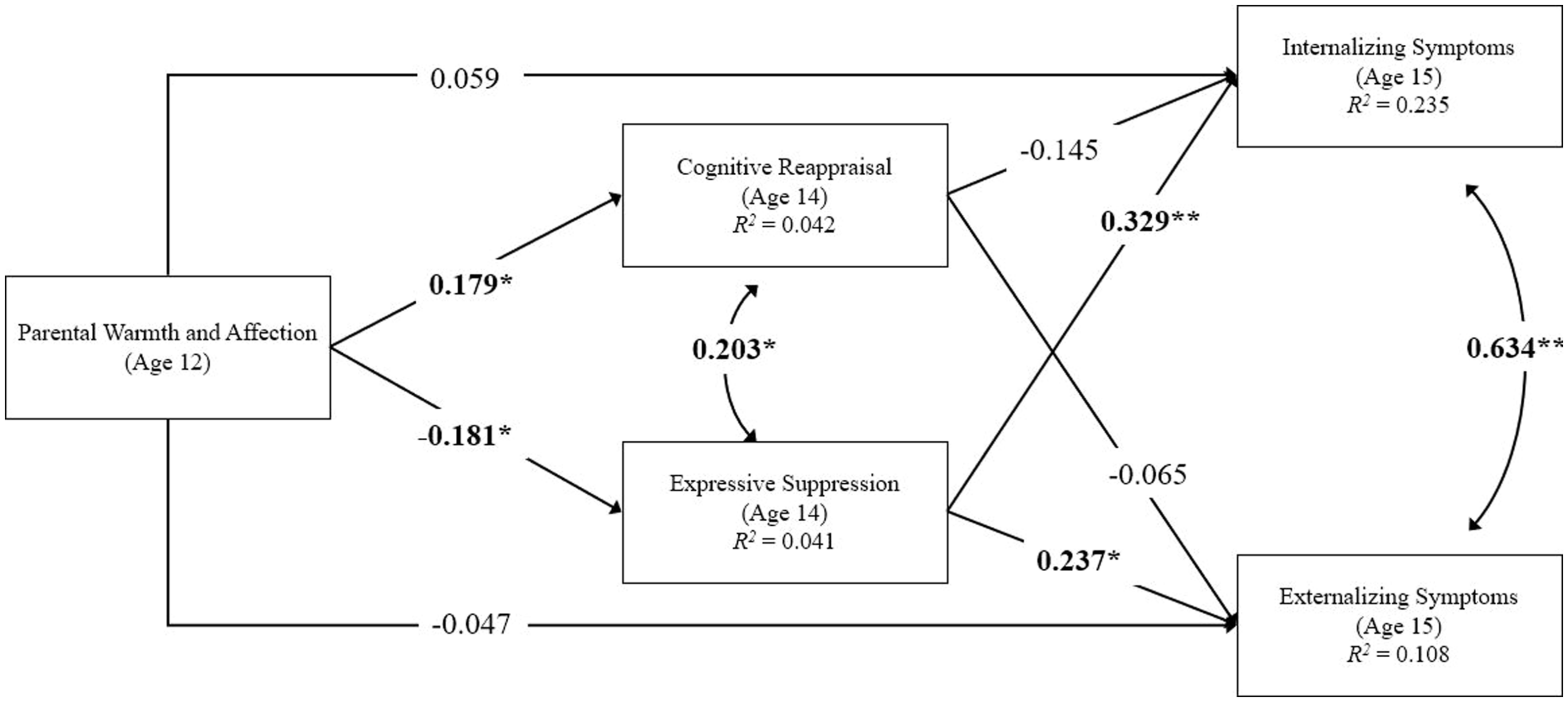
A parallel mediation model with cognitive reappraisal and expressive suppression (age 14) as mediators of warm and affectionate parenting (age 12) on internalizing and externalizing symptoms (age 15). Pathways depict standardized coefficients with significant relations indicated in bold with asterisks. Covariates (not shown) include child sex, race and ethnicity, income-to-needs, prior internalizing and externalizing symptoms (age 14), and stressful events during COVID-19 (age 15). *p < 0.05 level. **p < 0.001.

A parallel mediation model tested relations between parental warmth and affection at age 12 and changes in adolescents’ internalizing and externalizing problems from age 14 (pre-pandemic) to age 15 (COVID-19) as mediated by CR and ES emotion regulation strategy use at age 14 while controlling for sex assigned at birth (female =1, male = 0), ethnicity and race (Latinx = 1, non-Latinx = 0), and family income-to-needs (see Figure 1). Table 2 depicts unstandardized and standardized bootstrapped estimates of the mediation results. The mediation model accounted for 23.5% of the variance in adolescents’ internalizing problems (Cohen’s *f*^2^ = 0.307) and 10.8% of the variance in their externalizing problems (Cohen’s *f*^2^ = 0.121). Despite the absence of a significant direct effect from early adolescents’ reports of parental warmth and affection to adolescents’ internalizing and externalizing responses to the COVID-19 pandemic, indirect paths through CR and ES were tested as per current recommendations (Hayes, 2009). Results revealed a significant indirect effect from parental warmth and affection in early adolescence to fewer internalizing problems in response to the COVID-19 pandemic 3 years later (i.e., beyond pre-pandemic internalizing problems) via adolescents’ lower reliance on ES as an emotion regulation strategy at age 14. This indirect pathway accounted for 21.5% of the variance in adolescents’ internalizing problems during the COVID-19 pandemic. Neither the indirect effect through CR to internalizing problems, nor indirect pathways to externalizing problems through CR or ES attained significance.

**Table 2:** Standardized and unstandardized mediation model estimates.

Variable	*B*	SE	*β*	*p*	95% bias corrected		LLCI	ULCI
Covariates								
Female Sex = > Cognitive Reappraisal			0.06	0.07	0.07	0.318	−0.12	0.16
Female Sex = > Expressive Suppression			0.00	0.07	0.00	0.997	−0.14	0.14
Female Sex = > COVID-19 Internalizing			1.97	1.76	0.08	0.264	−1.48	5.42
Female Sex = > COVID-19 Externalizing			0.75	1.74	0.04	0.667	−2.65	4.15
Latine = > Cognitive Reappraisal			0.02	0.07	0.02	0.751	−0.12	0.16
Latine = > Expressive Suppression			−0.08	0.07	−0.08	0.275	−0.22	0.06
Latine = > COVID-19 Internalizing			−0.62	1.71	−0.02	0.719	−3.97	2.74
Latine = > COVID-19 Externalizing			−0.98	1.64	−0.05	0.549	−4.19	2.23
Family Income-to-Needs = > Cognitive Reappraisal			0.01	0.02	0.04	0.569	−0.03	0.06
Family Income-to-Needs = > Expressive Suppression			0.02	0.11	0.06	0.377	−0.02	0.06
Family Income-to-Needs = > COVID-19 Internalizing			−0.50	0.59	−0.06	0.394	−1.66	0.65
Family Income-to-Needs = > COVID-19 Externalizing			−0.19	0.50	−0.03	0.705	−1.18	0.80
Pre-COVID-19 Internalizing = > COVID-19 Internalizing			0.04	0.09	0.04	0.623	−0.13	0.21
Pre-COVID-19 Externalizing = > COVID-19 Externalizing			0.02	0.07	0.02	0.748	−0.12	0.16
COVID-19 Stressors = > COVID-19 Internalizing			**1.42**	**0.31**	**0.33**	**0.000**	**0.82**	**2.02**
COVID-19 Stressors = > COVID-19 Externalizing			**0.75**	**0.32**	**0.20**	**0.018**	**0.13**	**1.37**
Predictor pathways								
Parental Warmth and Affection = > Cognitive Reappraisal			**0.22**	**0.11**	**0.18**	**0.038**	**0.01**	**0.43**
Parental Warmth and Affection = > Expressive Suppression			**−0.23**	**0.11**	**−0.18**	**0.032**	**−0.44**	**−0.02**
Cognitive Reappraisal = > COVID-19 Internalizing			−3.70	1.96	−0.16	0.058	−7.53	0.13
Cognitive Reappraisal = > COVID-19 Externalizing			−1.47	2.13	−0.07	0.492	−5.65	2.71
Expressive Suppression = > COVID-19 Internalizing			**8.21**	**1.91**	**0.33**	**0.000**	**4.48**	**11.94**
Expressive Suppression = > COVID-19 Externalizing			**5.24**	**1.98**	**0.24**	**0.008**	**1.35**	**9.13**
Direct effects								
Parental Warmth and Affection = > COVID-19 Internalizing			1.90	2.47	0.06	0.443	−2.95	6.75
Parental Warmth and Affection = > COVID-19 Externalizing			−1.33	2.28	−0.05	0.561	−5.80	3.15
Indirect effects								
Parental Warmth and Affection = > Cognitive Reappraisal = > COVID-19 Internalizing			−0.83	0.60	−0.03	0.168	−2.01	0.35
Parental Warmth and Affection = > Expressive Suppression = > COVID-19 Internalizing			**−1.90**	**0.96**	**−0.06**	**0.047**	**−3.78**	**−0.03**
Parental Warmth and Affection = > Cognitive Reappraisal = > COVID-19 Externalizing			−0.33	0.51	−0.01	0.520	−1.33	0.67
Parental Warmth and Affection = > Expressive Suppression = > COVID-19 Externalizing			−1.22	0.75	−0.04	0.103	−2.68	0.25
Total indirect effect			**−4.48**	**2.03**	**−0.14**	**0.035**	**−8.25**	**−0.30**
*R*^2^*_internalizing_* = 0.235; Cohen’s *f*^2^*_internalizing_* = 0.307								
*R*^2^*_externalizing_* = 0.108; Cohen’s *f*^2^*_externalizing_* = 0.121								

Note: SE, standard error; LLCI, lower limit confidence interval; ULCI, upper limit confidence interval. Female = 1. Latinx = 1. All analyses were conducted using 95% bootstrapped CI. Significant findings are indicated in bold.

### Sensitivity analysis

A sensitivity analysis evaluated the proposed model among the 122 cases with complete data at all waves (i.e., list-wise deletion). This analysis replicated the primary study finding of a significant indirect effect from parental warmth and affection at age 12 to a reduction in adolescents’ internalizing problems during the COVID-19 pandemic through lower reliance on ES at age 14 (*B* = −1.99, *SE* = 0.10, *p* = 0.045) but not through CR (*B* = −0.98, *SE* = 0.17, *p* = 0.17). All other pathways were consistent with the full sample analyses, with two exceptions. First, the pathway from parental warmth and affection to cognitive reappraisal was marginal in this subsample (*p* = 0.066), but significant in the full sample (*p* = 0.038). Second, the pathway from cognitive reappraisal to internalizing symptoms was significant in this subsample (*p* = 0.034), but marginal in the full sample (*p* = 0.058).

## Discussion

This investigation advances our understanding of parenting influences on adolescents’ mental health during the COVID-19 pandemic. We evaluated whether and how parental warmth and affection at age 12 predicted changes in adolescents’ internalizing and externalizing problems across the transition to the U.S. COVID-19 pandemic lockdown. As predicted, adolescents who reported higher levels of warm and affectionate parenting at age 12 also endorsed greater use of CR and less use of ES emotion regulation strategies at age 14. Although CR was marginally related to fewer internalizing, but not externalizing, problems in response to the pandemic disruptions of spring 2020, CR did not emerge as a significant mediator of parenting effects on adolescents’ mental health during the pandemic. In contrast, adolescents’ pre-pandemic reliance on ES predicted significant elevations in both internalizing and externalizing problems during the initial phase of the COVID-19 pandemic and mediation analyses revealed a significant indirect pathway from parental warmth and affection to fewer internalizing problems during COVID-19 through adolescents’ reduced reliance on ES. The absence of significant pathways from CR to adolescents’ mental health problems and the modest magnitude of the pathway from ES to externalizing problems precluded the emergence of significant indirect effects.

A wealth of research demonstrates that warm and affectionate parenting can facilitate positive adaptation to stressful life events for children (McLaughlin and Lambert, 2017) and adolescents (Howard and Medway, 2004) from all cultural groups (Khaleque, 2013), including in the context of the COVID-19 pandemic (Chang et al., 2021; Cohodes et al., 2021; Wang et al., 2021). Emotion regulation has long been thought to mediate such effects (Morris et al., 2017), though, to the best of our knowledge, the current study offered a novel test of this hypothesis in a relatively large sample of adolescents as they confronted the initial phase of the COVID-19 pandemic. Moreover, whereas studies of parenting, emotion regulation, and adjustment have typically focused on single facets of emotion regulation (e.g., ES *or* CR; Balan et al., 2017) as related to either internalizing *or* externalizing problems (e.g., Walton and Flouri, 2010; Weissman et al., 2021), this investigation offered a comprehensive picture of pathways to adolescents’ internalizing and externalizing problems via both CR and ES strategies using a single multiple mediation model. In doing so, this study revealed interesting patterns whereby ES, but not CR, accounted for significant variance in adolescents’ internalizing, but not externalizing, problems during the COVID-19 pandemic.

Adolescents who perceive their parents as warm and affectionate may feel more comfortable expressing emotions (Alegre et al., 2014) in ways that garner support for their positive coping with stressors (Cameron and Overall, 2018). In turn, reduced reliance on ES may alleviate the physiological and psychological strain of stress exposure (Gross and Cassidy, 2019), including during the COVID-19 pandemic (Ellis et al., 2020). The salience of ES as a mediator of parenting effects on internalizing problems in this study is consistent with prior cross-sectional work suggesting that insensitive parenting is linked to adolescents’ internalizing problems through their increased reliance on ES (Balan et al., 2017). These findings also extend initial data from the COVID-19 pandemic suggesting that stressors related to COVID-19 were related to increased internalizing problems among adolescents who used more ES (Weissman et al., 2021), as well as with research showing that adolescents who had difficulties regulating their emotions prior to the pandemic endorsed more stress and lower social support during the initial months of the pandemic (Essau and de la Torre-Luque, 2021). The moderate effect size of the obtained indirect pathway from parental warmth and affection to adolescents’ reduced internalizing symptomatology via decreased reliance on ES is magnified by the universal salience of parenting, emotion regulation, and adaptation in all families such that even a small effect would have substantial practical significance (Funder and Ozer, 2019).

Although warm and affectionate parenting was related to greater use of CR by adolescents prior to the pandemic, CR only marginally predicted fewer internalizing problems during the pandemic and did not emerge as a significant mediator of parenting effects on adolescents’ mental health. It may be that CR is less helpful during the initial phase of a crisis because it entails attending to unpleasant stimuli (Moore et al., 2008). It may also be that mid-adolescents have not yet mastered the ability to successfully utilize CR (Ford and Troy, 2019). Thus, as proposed by Sheppes and Gross (2011), these findings suggest that, rather than universally positive or negative, the effectiveness of specific emotion regulation techniques vary depending on the timing and emotional intensity of a stressor. One implication of this interpretation is that adolescents should be taught a large repertoire of emotion regulation strategies and skills for optimizing strategy selection to the unique demands of a given challenge.

Significant pathways to internalizing, but not externalizing, problems align with prior work examining emotion regulation and psychopathology. Pepping et al. (2016) observed a similar pattern in their study of adolescents’ mindfulness and adjustment outcomes, finding that ES, but not CR, mediated the relation between mindfulness and fewer problems of depression and anxiety, but not fewer externalizing problems. In a study of college students’ coping with ethnic and racial discrimination, Juang et al. (2016) found that ES predicted higher internalizing, but not externalizing, problems. The use of ES may be less relevant to externalizing behaviors than more specific aspects of emotion expression, such as direct (i.e., expressing feelings toward the antagonist) versus indirect (i.e., expression of emotion not directed at the antagonist) strategies, which appear especially relevant for understanding externalizing outcomes (Brinke et al., 2021).

The absence of a significant direct pathway from parental warmth and affection to adolescents’ internalizing and externalizing problems during the COVID-19 pandemic was unexpected. Given the unique context of COVID-19 as a potent threat to the health of self and others, this finding may point to multiple (and potentially counteracting) pathways from parenting to adolescents’ mental health during COVID-19. For example, given well-documented relations between positive parenting and adolescent empathy (Padilla-Walker and Christensen, 2011), some adolescents who perceived their parents as warm and affectionate may also have been more attuned to (and affected by) the distress of parents and others during COVID-19. Likewise, the protective function of warm and affectionate parenting may have been countered by contrasting processes wherein these youth may have experienced heightened anxiety due to worries or concerns about their parent’s susceptibility to dying or becoming disabled from COVID-19. Moving forward, it will be important for researchers to elucidate moderating factors to clarify when and for whom these processes hold.

### Strengths and limitations

This study evaluated explanatory relations among parenting, emotion regulation, and adolescents’ mental health during the initial phase of the U.S. COVID-19 pandemic. Drawing on a relatively large and sociodemographically diverse sample across three data waves, this study filled gaps in the current literature by examining adolescents’ internalizing *and* externalizing problems from age 14 (pre-pandemic) to age 15 (early pandemic in spring 2020) as predicted by adolescents’ reports of parental warmth and affection at age 12 and explained by adolescents’ use of both CR *and* ES emotion regulation strategies at age 14. Despite these strengths, the current findings should be evaluated in consideration of several limitations.

First, the current sample was representative of the Southern California region from which the participants were recruited beginning in 2006 (U.S. Census Bureau, 2007), with particularly valuable representation of Latine participants (i.e., 46.6% of participating adolescents). At the same time, however, the current sample did not reflect the broader ethnic and racial composition of U. S. at the time of the COVID-19 pandemic (U.S. Census Bureau, 2020). Further, the sizes of each ethnic-racial group in the broader sample were too small to support our evaluation of the proposed model within each group. Although supportive parenting practices, such as warmth and affection, demonstrate consistently positive relations with adolescent development across diverse cultural and experiential contexts (Masten et al., 2004; Khaleque, 2013), research has shown significant variation in the adaptive significance of other parenting facets across groups (e.g., authoritative parenting; Chao, 2001; physical punishment; Deater-Deckard et al., 1996). Thus, it will be important to test indirect pathways among parenting, emotion regulation, and adolescent adaptation using a larger and more diverse nationally representative probability sample in future research.

Second, this study focused on only two emotion regulation strategies, leaving many additional strategies (e.g., avoidance, rumination; McRae and Gross, 2020) and distinctions (e.g., direct versus indirect expression; Brinke et al., 2021) unexamined. Further, the reliability for ES was acceptable, but modest. Moving forward, researchers should examine additional emotion regulation techniques individually and potentially collectively using well-validated measures. For example, some data point to the additional explanatory power afforded by attending to a combination of emotion regulation approaches (e.g., profile analysis; van den Heuvel et al., 2020), rather than only to individual strategies.

Third, although this longitudinal design with pre-pandemic controls supported directional interpretations of these findings, we were not able to evaluate causal assertions fully in the absence of prior emotion regulation strategy use patterns at age 12 to support a fully cross-lagged model. Consistent with broader models of child effects (Bell and Chapman, 1986), adolescents who utilize certain emotion regulation strategies or who struggle with specific socioemotional difficulties may evoke different degrees of warm and affectionate parenting. Future investigations that include measures of all variables at all waves would be best suited to evaluate the likely bidirectionality of these relations and strengthen causal claims about parental influences on adolescents’ emotion regulation and resulting psychopathology in the face of major stressors.

Fourth, this investigation was limited to self-report measures, which may have inflated observed relations due to shared method variance or distorted them due to informant bias. In particular, the obtained pathway to internalizing, but not externalizing, problems may reflect known tendencies for adolescents to report their externalizing behaviors less accurately as compared to parent reports or clinician diagnoses (Penney and Skilling, 2012). Future research will benefit from evaluating the current explanatory model using observational data and multi-informant reports from parents, teachers, and/or clinicians.

Finally, the low stability of internalizing and externalizing problems from ages 14 (pre-pandemic) to 15 (early pandemic) was surprising. Although the COVID-19 pandemic restrictions demanded a shift to online data collection methods, prior data support the validity of online data collection using the YSR (Achenbach et al., 2004). This instability may reflect the shift from a 6-month to 2-week symptom period to capture early-pandemic behavior problems and/or true instability in symptom expression across the transition into the pandemic. However, post-hoc analyses examining symptom stability in YSR reports across ages 12, 14, and 15 in this sample indicated that the phone-based administration of the YSR at age 14 may have biased adolescents’ reports during this assessment. Whereas YSR administrations at ages 12 in the lab and 15 on-line during the pandemic showed strong stability for both internalizing [*r*(145) = 0.346, *p* < 0.001] and externalizing [*r*(145) = 0.478, *p* < 0.001] problems across a three-year period, stability values were modest for externalizing problems [*r*(150) = 0.202, *p* = 0.013] and nonsignificant for internalizing problems [*r*(150) = 0.095, *p* = 0.243] from ages 12–14, even though both administrations occurred prior to the pandemic and over a shorter period of time than ages 12–15. Indeed, the lowest stabilities were seen across the one-year period spanning from the pre-pandemic phone assessment at age 14 to the COVID-19 on-line assessment at age 15 in the current study.

## Implications and conclusion

Accumulating data points to ongoing and negative effects of the COVID-19 pandemic on adolescent development (Samji et al., 2022). As noted in the U.S. Surgeon General’s Advisory on youth mental health during the COVID-19 pandemic, it is imperative to understand how to support adolescents as they navigate this and future challenges. Extant research demonstrates that parenting is a strong and enduring influence on adolescents’ socioemotional development, one with heightened salience in stressful contexts, such as the COVID-19 pandemic. Moreover, parenting is readily modified by both parent- and family-centered approaches (Ryzin and Dishion, 2012).

This study illuminated the importance of warm and affectionate parenting for adolescents’ emotion regulation strategy use. Teaching adolescents to be more expressive about their emotional experiences and to rely less on ES as an emotion regulation strategy can help them to cope with some of the anxiety and distress associated with the COVID-19 pandemic (and other stressors). Prior intervention studies suggest that adolescents can be taught a range of emotion regulation strategies (Houck et al., 2016), as well as how to flexibly engage such strategies in ways that maximize their positive adaptation (Eadeh et al., 2021). As the COVID-19 pandemic presents new variants and new viruses gain traction (e.g., monkeypox), efforts to help parents create warm and affectionate relational environments that encourage adolescents’ emotional expression, or at least deter them from engaging ES strategies, can be mobilized to protect promote adolescents’ mental health.

## Data availability statement

The raw data supporting the conclusions of this article will be made available by the authors, without undue reservation.

## Ethics statement

The studies involving humans were approved by University of California, Riverside. The studies were conducted in accordance with the local legislation and institutional requirements. Written informed consent for participation in this study was provided by the participants’ legal guardians/next of kin.

## Author contributions

TMY contributed to conception and design of the study. AMB organized the database. AMB and LK performed the statistical analysis. AMB wrote the first draft of the manuscript. All authors contributed to manuscript revision, read, and approved the submitted version.

## Funding

This investigation was supported by the National Science Foundation (DLS 1628820), The National Institutes of Health (R03 HD097623-01), and the UC Irvine COVID-19 Basic, Translational and Clinical Research Funding Opportunity. We extend our deepest appreciation to the families and youth who participate in the Child Representation and Regulation Project.

## Conflict of interest

The author declares that the research was conducted in the absence of any commercial or financial relationships that could be construed as a potential conflict of interest.

## Publisher’s note

All claims expressed in this article are solely those of the authors and do not necessarily represent those of their affiliated organizations, or those of the publisher, the editors and the reviewers. Any product that may be evaluated in this article, or claim that may be made by its manufacturer, is not guaranteed or endorsed by the publisher.
